# Limitations on the Detection Rate of High-Risk HPV by Hybrid Capture 2 Methodology in High Grade Intraepithelial (HSIL) or Atypical Squamous Cells-Cannot Exclude HSIL (ASC-H) Cytological Lesions with Proved CIN2+

**DOI:** 10.1155/2015/746502

**Published:** 2015-09-02

**Authors:** Jean-Christophe Noël, Philippe Simon

**Affiliations:** Unit of Gynecopathology, Erasme University Hospital-ULB, Brussels, Belgium

## Abstract

Recent literature data suggest that the high-risk human papillomaviruses (HR-HPVs) testing with several molecular techniques could be an alternative to cytology in the detection of cervical intraepithelial neoplasias of grade 2 or worse (CIN2+). However, any molecular techniques have its own limits and may give false negative results which must be clearly known before undertaking a primary HPV screening. This study aims to evaluate the performance of the high-risk HPV hybrid capture II detection kit (HCII) which is considered as a “gold standard technique” in a series of 100 women having proved both cytological lesions of atypical squamous cells-cannot exclude an HSIL (ASC-H) or high-grade squamous intraepithelial lesion (HSIL) and histological lesions of CIN2+. The clinical sensitivity of HCII in women with a cytological diagnosis of ASC-H/HSIL and a diagnosis of CIN2+ is high but not absolute and estimated at 96% (95,6% and 100% of women with a diagnosis of CIN2/3 or invasive squamous cell carcinoma, resp.). These data although they are infrequent must be clearly referred before to start an HPV primary screening of CIN2+ especially with HCII methodology.

## 1. Introduction

In Belgium, invasive cervical cancer is the 12th most common cancer type in women and about 600 new cases are diagnosed annually [1]. In our country, as in others, “high-risk HPVs” (HR-HPVs) (HPVs: 16, 18, 31, 33, 35, 39, 45, 51, 52, 56, 58, and 59) are clearly associated with invasive cervical cancers and preinvasive neoplastic intraepithelial lesions (CIN: cervical intraepithelial neoplasia) [2–5]. In addition to these HR-HPVs, eight other HPV types (26, 53, 66, 67, 70, 73, and 82) have also been classified as probably or possibly carcinogenetic in recent studies [5]. The evidence of the role of HR-HPVs in the genesis of cervical cancer brought the Belgian health care experts to consider that the HR-HPV testing could be an alternative to the cytology in the detection of CIN2+ lesions in particular in women over 30 years [1]. Indeed, in agreement with several randomized studies, HR-HPVs detection is more sensitive than cytology in the detection of CIN2+ lesions and has superior efficacy to lower the incidence of invasive cervical cancer [1, 6–10]. If this hypothesis of the screening of HR-HPVs instead of cytology in the detection of CIN2+ is attractive, it should not obscure the fact that any molecular technique for HR-HPVs detection has its own limits and may give false negative results [10]. Of course, these limits must be clearly known before undertaking a primary HPV screening but nevertheless these pieces of information are starved in the Belgian French-speaking part. In order to clarify these data, we decided to analyze the percentage of HR-HPVs positivity in lesions proven both on the one hand to be cytologically high-grade squamous intraepithelial lesions (“HSIL”) or atypical squamous cells-cannot exclude HSIL (ASC-H) and in the other hand to be associated with histological lesions of CIN2+. We used the Hybrid Capture 2 test (HC2, Digene Corporation Gainthersburg, USA) which is considered as one of the possible molecular “standard” techniques of HR-HPV [11].

## 2. Material and Methods

### 2.1. Sample Collection, Cytology, and p16 Immunohistochemistry

**Figure 1:**
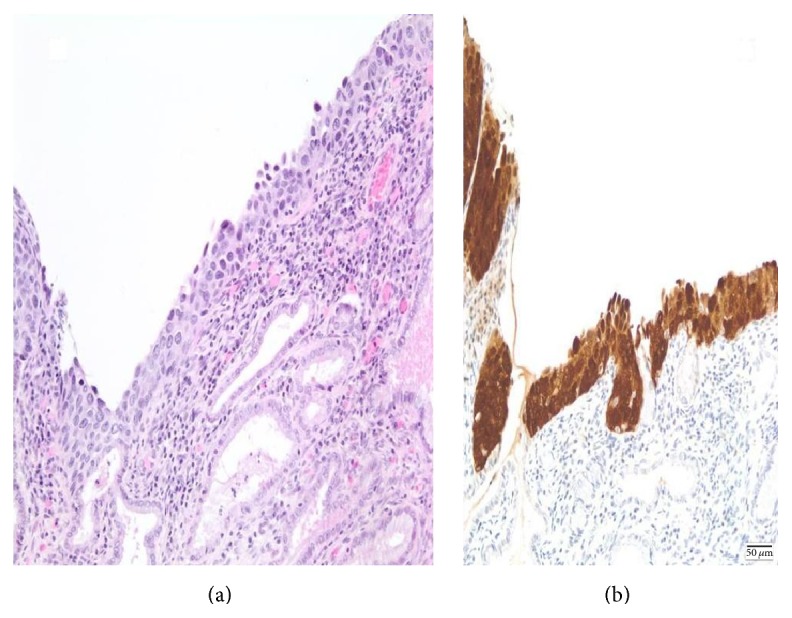
Typical example of CIN2/3 lesion HPV-negative (H&E) (a). Note the strong nuclear positivity for p16 protein (b).

This study was performed according to the rules of the Erasme Hospital's Ethics Committee (ethics committee approval references: P2015/234). 100 consecutive patients with a proven HSIL (*n* = 62) or ASC-H (*n* = 38) on cytology and CIN2+ lesions on biopsy (90 CIN 2/3 lesions and 10 invasive carcinomas/IC) were included in the study. All the patients (medium age: 42 years old; range: 25–77) were living in either Wallonia or Brussels (“Belgian French Community”). For the cytology, as previously published, we used a thin-layer technology (ThinPrep, Cytyc Corp., Boxborough, USA) from 20 mL vials that contained the collected cells (PreservCyt, Cytyc Corp., Boxborough, USA) as we have previously described [3, 12, 13]. The criteria used for the cytological and histological diagnosis were those reported, respectively, by the Bethesda System for reporting cervical cytology and by the WHO classification of tumor of female reproductive organs [14–16]. In addition, to exclude mimics of CIN2/3 lesions including immature squamous metaplasia, inflammation, or atrophy, an adjunctive p16 immunohistochemistry was also applied for each biopsy as previously published and was strongly positive in all the cases (Figure 1) [16].

### 2.2. Hybrid Capture 2 Assay

For HPV detection we used the Hybrid Capture 2 test with RNA probe cocktail for the following HR-HPV types 16, 18, 31, 33, 35, 39, 45, 51, 52, 56, 58, 59, and 68 from a residual quantity of 4 mL of PreservCyt vial after cytology as we have previously described [12, 13, 17]. In this study, the HC2 test was interpreted as positive when the ratio of relative light units (RLU/CO) to the positive control specimen was >1.0.

## 3. Results

96 of the 100 (96%) of HSIL/ASC-H cytological lesions with a CIN2+ lesion on biopsy were HR-HPV-positive with HC2. From them, 10 cases with a histological diagnosis of invasive carcinoma (ISCC) were positive (100%). In ASC-H or HSIL (*n* = 90) with CIN2/3 lesions on biopsy a positivity was observed in 86 cases (95,6%). The results are summarized in Table 1.

## 4. Discussion

The fact that firstly HR-HPV infection is a necessary cause for the development of CIN2+ lesions and secondly that DNA HPV testing has a higher sensitivity and reproducibility than cytology has led several countries including Belgium to the fact that a switch from cytology to HR-HPV detection could constitute an interesting alternative for cervical cancer screening [1, 2, 6–10]. In Belgium, it has been proposed that a technique to be validated for the primary screening should have a sensitivity at least equivalent to that from the GP5+/6+ PCR-based enzyme immunoassay (PCR EIA) (EIA: Diassay, Rijswijk, Netherlands) or HCII assay [1]. Indeed, these two tests have been extensively clinically validated particularly in the triage of borderline cytological lesion for colposcopy and have been considered, perhaps erroneously, as “references gold standard” [11]. However, in our country, at least in its French part, we do not have clear data regarding potential false negatives that might be caused by these techniques if they were used as a primary screening. In preliminary studies, particularly optimistic, it was suggested that the sensitivity of HCII assay for detecting histologically confirmed high-grade squamous intraepithelial lesions (CIN 2/3) is 100% [18]. In our work, we do not share this view because we have clearly demonstrated that at least 4% of women with highly atypical cytological lesions (ASC-H or HSIL) with CIN2+ diffusely immunoreactive for p16 protein are clearly negative by HCII test. Despite the fact that the sensitivity and specificity of p16 are not perfect, our results are consistent with recent literature data confirming that the sensitivity of the most widely validated tests for use in primary screening in the detection of CIN2+ lesions such as HCII test, GP5+/6+ PCR EIA assay, Cobas 4800 PCR assay (Roche molecular systems, Inc., Alameda, CA, USA), or Abbott real-time high-risk HPV PCR (Abbott Molecular, Des Plaines, IL, USA) is variable ranging from 95 to 97% (for the more optimistic studies and so forth) [11, 19–21]. The cause of the lack of “absolute” sensitivity in the detection of CIN2+ lesions is probably multiple and includes the sensitivity of the technique itself, the possibility that the regression of HPV infection may precede the regression of histological lesions or simply that the HPV causing CIN2+ lesions is not present in the various HPV cocktail provided in the different tests [5, 11]. However, it is interesting to note that, in this study, all cases of ISCC with a positive cytology were detected by HCII methodology, which is reassuring information altogether. Obviously, our study has limitations, firstly because the CIN2 or 3 lesions have not been differentiated and secondly because mainly not only CIN2 but also CIN3 may regress.

In conclusion, HPV primary screening has a significantly better sensitivity (>90% even 95%) than cytology and appears as a future robust methodology for a large and rapid screening. Nevertheless, contrary to a conventional wisdom, we demonstrated that, in our population, there were high-grade or suggested high-grade cytological abnormalities with clearly proven CIN2+ lesions for which the research of viral HR HPVs sequences with HCII methodology was negative. These data, although they are infrequent, must be known and referred before to start an HPV primary screening of CIN2+ lesions especially with HCII methodology which is considered by many as a reference standard technology particularly in Belgium.

## Figures and Tables

**Table 1:** Correlations between cytological and histological diagnosis and HR-HPVs positivity with HCII methodology.

Cytological diagnosis	Histological diagnosis	HR-HPVs positivity with HCII methodology
ASC-H (*n* = 32)	CIN2/3	31 (97%)
ASC-H (*n* = 6)	ISCC	6 (100%)
HSIL (*n* = 58)	CIN2/3	55 (95%)
HSIL (*n* = 4)	ISCC	4 (100%)

**ASC-H or HSIL (** **n** = 100**)**	**CIN2+**	**96 (96%)**
